# Distinctive Personality Traits and Neural Correlates Associated with Stimulant Drug Use Versus Familial Risk of Stimulant Dependence

**DOI:** 10.1016/j.biopsych.2012.11.016

**Published:** 2013-07-15

**Authors:** Karen D. Ersche, P. Simon Jones, Guy B. Williams, Dana G. Smith, Edward T. Bullmore, Trevor W. Robbins

**Affiliations:** a University of Cambridge, Behavioural and Clinical Neuroscience Institute, Departments of Experimental Psychology and Psychiatry, Cambridge, United Kingdom; b Wolfson Brain Imaging Centre, University of Cambridge, Cambridge, United Kingdom; c GlaxoSmithKline, Clinical Unit Cambridge, United Kingdom; d Cambridgeshire & Peterborough National Health Service Foundation Trust, Cambridge, United Kingdom

**Keywords:** Cocaine, compulsivity, familial vulnerability, impulsivity, resilience, sensation-seeking

## Abstract

**Background:**

Stimulant drugs such as cocaine and amphetamine have a high abuse liability, but not everyone who uses them develops dependence. However, the risk for dependence is increased for individuals with a family history of addiction. We hypothesized that individuals without a family history of dependence who have been using cocaine recreationally for several years but have not made the transition to dependence will differ in terms of personality traits and brain structure from individuals who are either dependent on stimulants or at risk for dependence.

**Methods:**

We compared 27 individuals without a familial risk of dependence who had been using cocaine recreationally with 50 adults with stimulant dependence, their nondependent siblings (*n* = 50), and unrelated healthy volunteers (*n* = 52) who had neither a personal nor a family history of dependence. All participants underwent a magnetic resonance imaging brain scan and completed a selection of personality measures that have been associated with substance abuse.

**Results:**

Increased sensation-seeking traits and abnormal orbitofrontal and parahippocampal volume were shared by individuals who were dependent on stimulant drugs or used cocaine recreationally. By contrast, increased levels of impulsive and compulsive personality traits and limbic-striatal enlargement were shared by stimulant-dependent individuals and their unaffected siblings.

**Conclusions:**

We provide evidence for distinct neurobiological phenotypes that are either associated with familial vulnerability for dependence or with regular stimulant drug use. Our findings further suggest that some individuals with high sensation-seeking traits but no familial vulnerability for dependence are likely to use cocaine but may have relatively low risk for developing dependence.

According to estimates by the United Nations, approximately 21 million people worldwide are using cocaine (1), but only one in six cocaine users appears to make the transition from occasional cocaine use to cocaine dependence (2). Although the likelihood of becoming dependent on cocaine is increased in people with a family history of drug/alcohol dependence (3), this risk is not reflected in contemporary categorizations of cocaine users. Recreational users are usually described as socially integrated people who use cocaine infrequently, in small amounts at social occasions, without experiencing psychological or physiological signs associated with cocaine abuse (4–6). Problem or dependent cocaine users, however, use cocaine in a more habitual manner that is associated with physical, social, and psychological problems, for which the user seeks help (4,5). Generally, these individuals satisfy the criteria for a clinical diagnosis of cocaine dependence stipulated by the American Psychiatric Association (7), but for recreational users, no such criteria are available. Whether recreational and dependent cocaine users reflect different neurobiological phenotypes or are expressing variable degrees of impairment on the same phenotype along a trajectory to dependence warrants investigation.

Preclinical evidence supports the notion that different cocaine user types can be distinguished by traits of sensation-seeking (predicting the initiation of cocaine intake) and impulsivity (predicting the development of compulsive cocaine seeking and dependence) (8). Analogously, impulsive traits and ritualistic behavior tendencies in human cocaine users have been associated with a familial risk for developing dependence (9–11) and sensation-seeking traits have been associated with a risk of initiating drug use during adolescence (12,13). We sought to illuminate the neurobiological basis of recreational cocaine use further by comparing four groups of individuals who differed with regard to two factors: familial risk for addiction and stimulant drug exposure. Specifically, we compared 27 individuals without a familial risk of dependence who had been using cocaine for an average of 8 years (±5.9 SD) but had not made the transition to dependence with a previously published sample of 1) 50 individuals who satisfied the DSM-IV criteria for dependence on stimulant drugs; 2) 50 unaffected siblings of the stimulant-dependent individuals; and 3) 52 unrelated healthy volunteers who had neither a family nor a personal history of dependence. We compared these four groups in terms of sensation-seeking traits, impulsivity, ritualistic behavior (a possible marker of compulsive traits), and brain structure.

Abnormalities in corticostriatal pathways, such as a relative reduction in prefrontal cortex gray matter and an increase in striatal volume, have been associated with impulsive and compulsive traits and have frequently been reported in stimulant-dependent individuals (14–19). First-degree relatives share with their dependent family member the striatal enlargement (in the putamen) but not the gray matter deficits in the prefrontal cortex (20), indicating that the reduced prefrontal volume is not a candidate endophenotype predisposing stimulant dependence but may be a consequence of chronic stimulant abuse. If recreational cocaine users constitute a different neurobiological phenotype from dependent stimulant users or their at-risk first-degree relatives, we would expect their levels of impulsivity and obsessive-compulsive behaviors, as well as their striatal structure, to be normal. However, if recreational cocaine users exhibit a trajectory toward dependence, we would expect to identify abnormalities, specifically in the prefrontal cortex, that are typically seen in stimulant-dependent individuals, albeit to a lesser degree.

## Methods and Materials

### Study Sample and Procedures

We recruited 27 individuals by advertisements in the local community who fulfilled the following criteria: 1) no personal or family history of substance dependence (including alcohol but excluding nicotine); 2) repeated use of cocaine for at least 2 years without experiencing physiological or psychological symptoms of dependence, as described in the DSM-IV; and 3) no use of stimulant drugs for medical reasons. Exclusion criteria were a lifetime history of a psychiatric or neurological disorder, neurodevelopmental disorder, or a traumatic head injury. These individuals were recruited in addition to a previous sample of participants (20) consisting of 50 biological sibling pairs (within each pair, one sibling satisfied the DSM-IV text revision [DSM-IV-TR] criteria for dependence on stimulant drugs and the other had no history of drug or alcohol dependence) and 52 unrelated healthy volunteers with no personal or family history of drug/alcohol dependence. Tobacco smokers were not excluded from the study to ensure variation of smoking habits across groups.

The recreational cocaine users started using cocaine at the age of 21 years (±5.1 SD) and had used it in relatively small amounts (mean .6 g±.3 g SD illicit drug dose) infrequently ever since. They exclusively used cocaine in powdered form in social settings with friends and never developed patterns of compulsive use, which was reflected by their low scores (mean 1.2±1.6 SD) on the Obsessive-Compulsive Drug Use Scale (21). Almost all recreational cocaine users (96%) had a lifetime history of sporadic experimentation with other illicit drugs than cocaine but never fulfilled the DSM-IV-TR criteria of substance dependence or ever considered seeking treatment for drug or alcohol use. Accordingly, their scores on the Drug Abuse Screening Test (DAST-20) (22) and the Alcohol Use Identification Test (AUDIT) (23) were low (Table 1).

All drug-dependent individuals met the DSM-IV-TR criteria for stimulant dependence (94% cocaine, 6% amphetamines). The majority was recruited from treatment services (76%) and all except five were actively using stimulant drugs by nasal, oral, or intravenous routes. They started using stimulants at the age of 16 years (±2.8 SD) and had been using them in varying amounts for an average of 16 years (±6.4 SD). Their Obsessive-Compulsive Drug Use Scale scores indicated moderate levels of stimulant-related compulsivity (mean score: 23.7±9.5 SD). All stimulant-dependent individuals were regularly using other substances alongside stimulants; 54% of the sample met the DSM-IV-TR criteria for dependence on opiates, 24% met the criteria for dependence on alcohol, and 8% met the criteria for dependence on cannabis. Their biological siblings were screened for drug and alcohol use, but none of the siblings met criteria for substance dependence as outlined in the DSM-IV-TR . Seventy-six percent of the siblings reported recreational use of cannabis, which was also mirrored in notably low scores on the DAST-20 and the AUDIT (Table 1). None of the healthy volunteers reported taking prescribed or illicit drugs on a regular basis, but 21% reported having used cannabis. None of the healthy volunteers had a lifetime history of drug dependence, according to the DSM-IV-TR criteria. Urine sampled on the testing day was positive for stimulants for all stimulant-dependent individuals except five individuals and negative for all recreational cocaine users, siblings, and healthy control volunteers tested for standard illicit substances, including cocaine, amphetamines, methamphetamine, ecstasy, and opiates.

### Assessment Procedures

All participants followed the same protocol, as described elsewhere (20). In brief, they were screened for any other current Axis I psychiatric disorder using the Structured Clinical Interview for the DSM-IV-TR Axis I Disorders (24). They underwent a semistructured interview to ascertain history of drug use, physical health, including signs of acute intoxication and withdrawal, and completed the Beck Depression Inventory Second Edition (25) to assess depressive mood. We used the National Adult Reading Test (26) to estimate their verbal IQ and the Childhood Trauma Questionnaire (27) to assess traumatic childhood experiences. All participants completed the Barratt Impulsiveness Scale version 11 (28) to measure impulsivity, the Padua Inventory-Washington State University Revision (29) to evaluate obsessive-compulsive tendencies, and the Sensation-Seeking Scale-Form V (30) to assess sensation-seeking traits. We also administered the DAST-20 and the AUDIT to quantify participants’ drug and alcohol use, as both measures have demonstrated sensitivity in nonclinical populations. The study protocol received ethical approval from the Cambridge Research Ethics Committee (REC08/H0308/310; Principal Investigator: K.D. Ersche) and written informed consent was obtained from all participants before study enrollment.

### Acquisition of the Neuroimaging Data

Scanning was performed at the Wolfson Brain Imaging Centre, University of Cambridge, United Kingdom, using a Siemens TIM-Trio 3T system (Siemens, Erlangen, Germany). Whole-brain T1-weighted magnetic resonance scans were acquired first from the control volunteers and the sibling pairs, as previously described (20), and subsequently from the recreational users using a magnetization prepared rapid acquisition gradient-echo sequence (176 slices of 1 mm thickness, repetition time = 2300 msec, echo time = 2.98 msec, inversion time = 900 msec, flip angle = 9°, field of view = 240×256). The magnetization prepared rapid acquisition gradient-echo images were segmented to produce modulated gray matter density images in standard Montreal Neurological Institute space, followed by smoothing with an isotropic Gaussian kernel of 2.35 mm using the FSL-VBM (http://www.fmrib.ox.ac.uk/fsl/fslvbm/index.html, version 4.1; Oxford Centre for Functional MRI of the Brain, Oxford, United Kingdom) pipeline. The 179 participants who completed the study were enrolled in one of the following four groups: recreational cocaine users, stimulant-dependent individuals, the biological siblings of the dependent users, and unrelated healthy volunteers. Magnetic resonance imaging brain scans for three stimulant-dependent individuals (two of whom provided stimulant-negative urine samples), one sibling, and one recreational user were unavailable, leaving the final sample constituted as follows: recreational users (*n* = 26), stimulant-dependent individuals (*n* = 47), siblings (*n* = 49), and healthy volunteers (*n* = 52).

### Statistical Analysis

Demographic and psychometric data were analyzed using SPSS v19 (IBM Corp., Armonk, New York). The PI-WRSU total scores were square-root transformed to reduce skew (31). To improve comparability of the three personality traits, we normalized the psychometric data with respect to the mean and standard deviation of each trait score in the healthy control group. Group comparisons were performed using analysis of covariance models with gender as a covariate, followed by post hoc comparisons corrected by Bonferroni methods. We initially considered including childhood trauma, verbal IQ, years of education, and alcohol use (AUDIT score) as covariates in the analysis to control for group differences (Table 1) but all four variables interacted with group status, suggesting that these are defining features of the groups. Consequently, co-varying for them would not be appropriate (32). We did not consider including age as a covariate in the analysis because the groups did not significantly differ in age. Moreover, the inclusion of age would have been inappropriate because there is evidence that chronic cocaine use interacts with age-related gray matter decline (33). Subsequent analyses with both tobacco and cannabis smoking status as covariates were conducted to verify no confounding influences on the results. All tests were two-tailed and a significance level of .05 was assumed.

**Figure 1:**
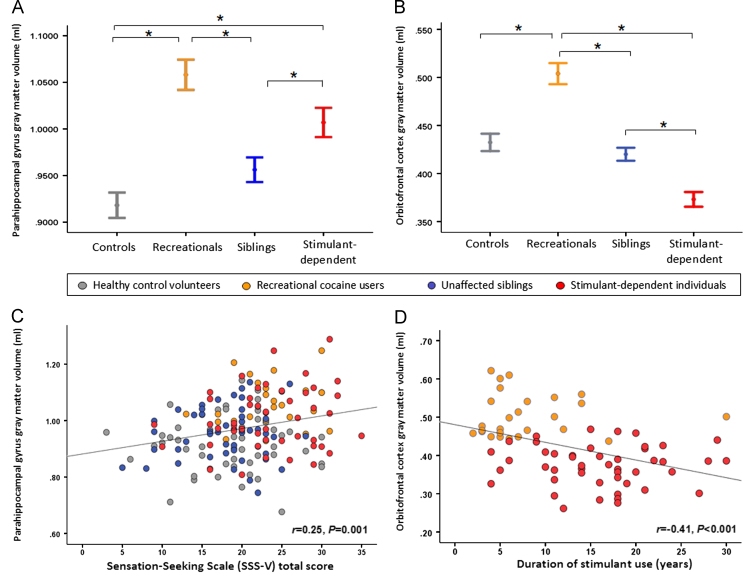
Abnormalities in gray matter volume in recreational cocaine users and stimulant-dependent individuals (identified by comparisons with healthy control volunteers) were overlapping in the parahippocampal gyrus and the orbitofrontal cortex. **(A)** Parahippocampal volume was significantly increased in both drug user groups compared with control volunteers and also compared with the siblings. **(B)** The group difference in gray matter volume in the orbitofrontal cortex was due to recreational users showing a significant volume increase compared with healthy control volunteers and siblings, whereas orbitofrontal volume in the stimulant-dependent volunteers was significantly reduced compared with the other three groups. **(C)** Gray matter volume in the parahippocampal gyrus was associated with levels of sensation-seeking personality traits in all volunteers. **(D)** Relationship between orbitofrontal gray matter volume and stimulant use in recreational and dependent users: the longer individuals have been using stimulant drugs, the greater the decline in orbitofrontal volume. ^⁎^Significant post hoc comparisons following Bonferroni correction. SSS-V, Sensation-Seeking Scale-Form V.

All magnetic resonance images were screened for abnormal radiological appearance by a specialist in neuroradiology and were analyzed using FSL-VBM, as described elsewhere (20). To identify abnormalities of gray matter, two-sample *t* tests were performed on the gray matter maps in CamBA software version 2.3.0 (http://www-bmu.psychiatry.cam.ac.uk/software/; Brain Mapping Unit, University of Cambridge, United Kingdom) using permutation testing of cluster mass (34) with 32 randomizations. The expected number of false-positive clusters was set at less than one. The whole-brain statistical maps for each test were thresholded at cluster level of≤1 error clusters per image and had equivalent *p* values: control volunteers ≅ recreationals, *p* = 7×10^−4^; control volunteers ≅ siblings, *p* = 3×10^−4^; and control volunteers ≅ drug users, *p* = 3×10^−4^. The independent sample *t* tests were performed to compare the brains of recreational users, stimulant-dependent individuals, and their siblings with those of control volunteers. Separate group comparisons limited to either male or female gender did not change the results. A region of interest analysis was subsequently performed on overlapping clusters from the different *t* tests. The mean gray matter volumes from individuals in all four groups were extracted from the regions of abnormalities common to both recreational and dependent users (labeled by the Hammers probabilistic atlas [35]), imported into SPSS v19 for correlational analysis (i.e., gray matter and sensation-seeking and obsessive-compulsive traits), and used to produce Figure 1.

## Results

### Demographics and Personality Traits

The demographic characteristics of the four groups are shown in Table 1. Due to the male dominance in the stimulant-dependent sample, the four groups differed significantly with regard to gender (χ^2^ = 18.6, *p*<.001). Treatment-seeking and nontreatment-seeking dependent users did not differ in demographics, personality, or clinical measures. The sibling pairs had significantly higher levels of trauma compared with healthy control volunteers [*F*(3,174) = 12.3, *p*<.001; post hoc both *p*<.05]. Levels of trauma in stimulant-dependent individuals were also significantly higher compared with recreational cocaine users (post hoc, *p*<.001) but not from their siblings (post hoc, *p* = .128). We further observed differences between the groups with regard to verbal IQ [*F*(3,163) = 4.1, *p* = .008], the duration of formal education [*F*(3,175) = 5.8, *p* = .001], their disposable income [*F*(3,173) = 3.0, *p* = .032], and marginally for age [*F*(3,175) = 2.4, *p* = .073]. These differences were due to the fact that the stimulant-dependent group had spent less time in education compared with both the healthy volunteers and the recreational users (both *p*<.05). The recreational users showed significantly higher levels of verbal IQ compared with the siblings (*p* = .008) and were also marginally significantly higher compared with the stimulant-dependent individuals (*p* = .076). They also had more money to spend compared with the drug-dependent individuals (*p* = .043), but the comparison with the disposable income of the siblings did survive correction for multiple comparisons.

**Figure 2:**
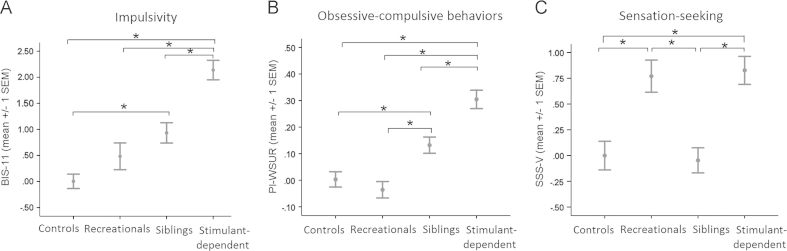
Personality traits associated with stimulant dependence, shown as *Z* scores. Familial risk in both stimulant-dependent individuals and their unaffected siblings was associated with significantly increased levels of both **(A)** impulsivity and **(B)** ritualistic behaviors. **(C)** Stimulant use, either recreationally or chronically, was reflected by greater than normal levels of sensation-seeking traits compared with healthy control volunteers and siblings. ^⁎^Significant post hoc comparisons following Bonferroni correction. BIS-11, Barratt Impulsiveness Scale version 11; PI-WSUR, Padua Inventory-Washington State University Revision; SSS-V, Sensation-Seeking Scale-Form V.

As displayed in Figure 2A,B, the levels of impulsivity [*F*(3,174) = 23.6, *p*<.001; post hoc test: *p* = .580] and obsessive-compulsive tendencies [*F*(3,174) = 21.3, *p*<.001; post hoc test: *p* = 1.0] did not differ between recreational users and control volunteers. The recreational users’ traits stand in stark contrast to both stimulant-dependent individuals and their siblings, who both differed significantly from healthy volunteers in terms of impulsivity (both *p*≤.001) and obsessive-compulsive behaviors (both *p*<.05). With regard to sensation-seeking traits [*F*(3,174) = 9.6, *p*<.001], recreational and dependent users both exhibited higher than normal levels of sensation-seeking compared with healthy volunteers as well as siblings (post hoc tests; *p*≤.001; Figure 2C). Sensation-seeking levels in the siblings, however, were normal (post hoc test: *p* = 1.0). Subsequent removal of either the three individuals who were dependent on amphetamines or the five stimulant-dependent users who provided stimulant-negative urine samples did not change the results (see also Figure S2 in Supplement 1). Current treatment status did not affect the highly significant difference in impulsivity and compulsivity between recreational users and dependent users (all *p*≤.005), nor were there any additional differences in sensation-seeking traits (*p* = .596).

### Gray Matter Volume

**Figure 3:**
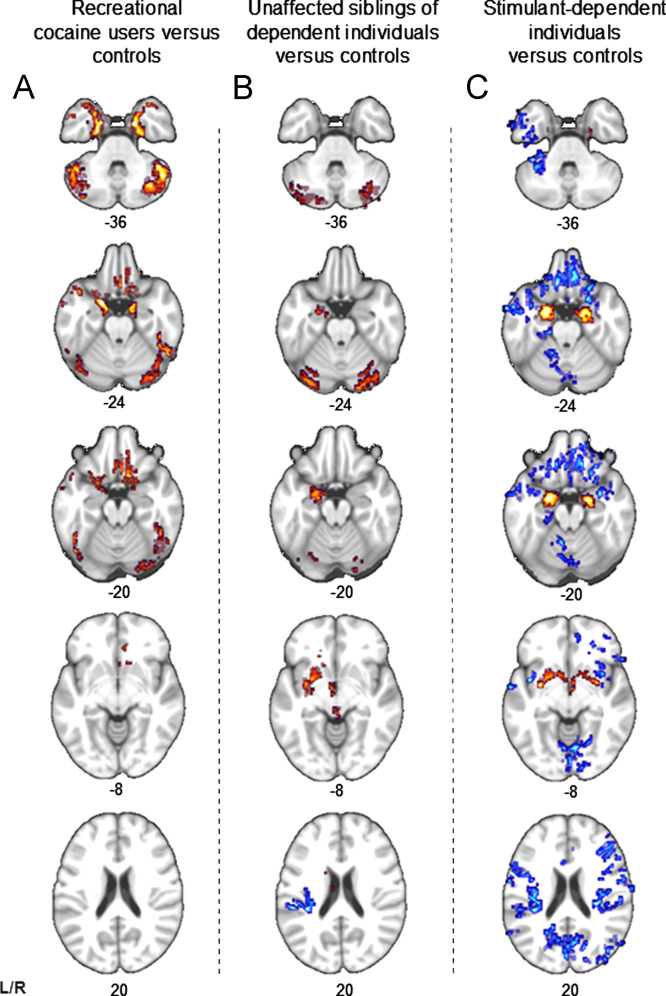
Structural abnormalities associated with stimulant exposure and familial risk. Blue voxels indicate a decrease and red voxels indicate an increase in gray matter volume compared with control volunteers. Both recreational and dependent stimulant users showed significant increase in the parahippocampal gyrus compared with healthy control volunteers but differed with regard to abnormalities in the orbitofrontal cortex. Recreational users did not show any of the changes in brain regions associated with familial risk such as increased volume of amygdala and putamen and decreased volume in posterior insula. L, left; R, right.

As shown in Figure 3A, the recreational user group did not exhibit any changes in brain structure associated with familial risk of dependence (20), i.e., their volumes of the amygdala, putamen, and posterior insula were normal. Recreational users, however, shared with the dependent users abnormally increased gray matter volume in the parahippocampus gyrus bilaterally, which extended to anterior parts of the amygdala and hippocampus (see Figures 1A and 3A). This volume increase in the parahippocampal gyrus was associated with sensation-seeking traits (*r* = .25, *p* = .001), displayed in Figure 1C. Both recreational and dependent users also showed abnormal orbitofrontal volumes but of a different nature: stimulant-dependent individuals showed significant volume reductions, whereas recreational users showed significantly increased orbitofrontal volume (Figure 1B). Orbitofrontal volume was negatively correlated with the duration of stimulant use (*r* =−.41, *p*<.001), displayed in Figure 1D, and with obsessive-compulsive tendencies (*r* =−.29, *p*<.001) in all volunteers. To verify the double dissociation between stimulant use and familial risk, we additionally compared the recreational group with the sibling group and the stimulant-dependent group; the results are shown in Figure S1 in Supplement 1. It is also of note that recreational users and siblings shared increased gray matter volume in the cerebellum (Figure 3).

## Discussion

We found generic and specific phenotypes associated with familial risk and stimulant use. As hypothesized, recreational users without a family history of dependence showed none of the previously observed endophenotypic markers of addiction, i.e., their levels of impulsivity and obsessive-compulsive tendencies were not increased and their brain structure did not show any changes that have been associated with familial risk (20). We did, however, observe similarities in personality traits and variations in brain structure that have been associated with stimulant dependence.

### Brain and Behavioral Characteristics Associated with Stimulant Use

Both recreational cocaine users and stimulant-dependent individuals reported higher than normal levels of sensation-seeking. These findings should not come as a surprise, given the relatively large number of research studies suggesting that sensation-seeking is a strong predictor of drug use among adolescents and adults (12,13,36–38). Factor analyses have further demonstrated that sensation-seeking traits discriminate between students who engage in drug taking and those who do not (39). Animal models of addiction also support the proposal that sensation-seeking traits are associated with the use of stimulant drugs (40). However, despite the strong relationship between sensation-seeking and drug taking, it is also important to note that the trait characterizes a need for stimulation that is expressed by a tendency to seek out novel experiences (41), which do not necessarily involve addictive drugs. Yet, converging lines of evidence suggest that exposure to novelty and addictive drugs may involve overlapping neural networks (42–45), which may explain why individuals who seek out novelty may also engage in drug-taking. More recent evidence indicates that novelty plays an important role in learning and memory (46). Medial temporal lobe structures, such as the parahippocampal formation, are thought to be critically involved in the anticipation of novelty (47,48), which may underlie the exploratory behavior in new environments seen in sensation-seeking individuals. Parahippocampal volume was significantly increased in both stimulant user groups and this volume increase was weakly but significantly associated with sensation-seeking traits. However, the functional implications of this structural abnormality for drug-taking behavior warrant further investigation, specifically in light of the evidence suggesting that the parahippocampal formation is also implicated in memory function (49) and cocaine craving, potentially related to cocaine-related memory retrieval (50,51).

We further identified in both stimulant user groups altered gray matter volumes in the orbitofrontal cortex, a region implicated in affective decision-making and goal-directed behaviors (52–54). Both reduced volume in the orbitofrontal cortex and dysfunctional decision-making in adolescence have been associated with the onset of substance abuse (55,56), possibly increasing the risk of developing dependence (57). Orbitofrontal function is known to be impaired in adults with cocaine dependence (58); specifically, disruptions in orbitofrontal functioning have been associated with compulsive features of stimulant drug dependence (59,60). Consistent with the contemporary literature, stimulant-dependent individuals showed significant reduction in gray matter volume in the orbitofrontal cortex (Figure 2C) (15,17,18,61–65), whereas the recreational users showed a significant increase in gray matter volume in this area (Figure 2A). Orbitofrontal volume decline has previously been associated with prolonged stimulant use (15,17), suggesting that the observed gray matter reduction reflects either a stimulant-induced effect or a nonfamilial vulnerability marker for stimulant dependence. As recreational users did not have the same extent of exposure to cocaine as the stimulant-dependent group, their abnormal increase in gray matter volume in this brain region might reflect an expression of resilience to the effects of cocaine and possibly reflect advantageous decision-making abilities or inhibitory control.

### Translational Evidence for Differential Personality Traits Associated with Stimulant Use

The differences in personality traits between recreational cocaine users and the sibling pairs are striking in light of animal models of addiction vulnerability. Rats exhibiting high levels of sensation-seeking traits, possibly as reflected by increased locomotor activity in novel environments, are fast learners in self-administering cocaine and tend to escalate their use when given free access to the drug (8,66,67). By contrast, impulsive rats, as identified by premature responses on the 5-choice serial reaction time task (68), do not show accelerated learning in cocaine self-administration (8). Yet, the way in which impulsive rats consume cocaine resembles patterns of compulsive cocaine-seeking in humans. Their persistence in the seeking of cocaine in the face of receiving response-contingent electric shocks may serve as a model for compulsive cocaine use seen in dependent individuals who continue using the drug despite aversive consequences precipitated by further use (8).

While impulsive personality traits seem to be a good predictor for dependence, the transition from recreational to dependent use does not occur overnight but develops in response to repeated drug exposure. An individual’s propensity to form habits may thus facilitate neuroadaptive changes underlying the transition (69,70). In the present study, we used the PADUA inventory (29) to assess individuals’ affinities for habits, mannerisms, and rituals, which in the case of obsessive-compulsive disorder become out of control (71). The significant increase in obsessive-compulsive behaviors in the sibling pairs, combined with their high levels of impulsivity, is likely to reflect their high risk for the development of dependence. Although the siblings shared the same vulnerability markers with their dependent brothers and sisters, they did not show increased levels of sensation-seeking traits. It is conceivable that their nonsensation-seeking personalities may have protected them from engaging in drug-taking behaviors. Individuals with high levels of sensation-seeking traits seem more likely to experiment with drugs, but if they have no familial vulnerability, they may have a low risk of developing dependence, despite continuous use.

### Implications of the Findings

Our findings indicate that recreational cocaine users without familial vulnerability are associated with a distinctive brain and behavioral phenotype that differs from the phenotype associated with cocaine dependence. Possibly, these characteristics represent an endophenotype for a relatively low risk for drug dependence despite continued exposure to cocaine. This hypothesis needs to be tested by examining the nondrug-taking siblings of the recreational users. Our findings demonstrate that the use of cocaine does not necessarily lead to addiction in individuals without familial risk who start using the drug after puberty, but it remains elusive as to whether the recreational users suffered any form of negative effects resulting from their cocaine use. Although we asked about negative consequences of cocaine use, recreational cocaine users reported none, but stressed their hedonistic motives for using cocaine, which they solely consumed together with friends at planned occasions. It is of note that cocaine use was only one of many pleasurable leisure activities they regularly exhibited, and they reported taking great care in preventing the drug from interfering with their personal goals. The role of personal life goals in regulating drug-taking activities might be of interest, given that concerns about such interference were among the most cited reason of students in the Drug Abuse Resilience Survey for abstaining from drugs (72). The recreational users’ success in pursing their goals despite the continuous use of cocaine might reflect exceptional capacities for self-regulation, which may be subserved, in part, by the orbitofrontal cortex (54,73). This may also be reflected in the negative relationship we observed between obsessive-compulsive tendencies and orbitofrontal volume.

Finally, the results further indicate that preventative strategies need to be tailored to suit dependent rather than recreational users. Interventions might be more effective if focused on young users whose neurobehavioral profile and brain structure predict that they are most likely to become dependent if they continue use. Our findings demonstrate that the use of cocaine does not inevitably lead to addiction in individuals without familial risk who start using the drug after puberty, but it remains elusive as to whether the recreational users suffered any form of negative effects resulting from their cocaine use. Further longitudinal studies will be required to evaluate the natural history of recreational cocaine use more extensively.

## Figures and Tables

**Table 1:** Demographic Information About the Four Groups

	Healthy Control Volunteers		Recreational Cocaine Users		Unaffected Siblings		Stimulant-Dependent Siblings		Group Comparisons		
	(*n* = 52)		(*n* = 27)		(*n* = 50)		(*n* = 50)				
Demographics	Mean (±SD)		Mean (±SD)		Mean (±SD)		Mean (±SD)		*F*, *t*, or χ^2^*p*		Post Hoc Tests^a^

Gender (% Male)	64%		52%		50%		88%		18.6	<.001	D>C, R, S
Age (Years)	32.5	(±8.9)	29.1	(±7.6)	32.9	(±8.4)	34.3	(±7.2)	2.4	.073	
Verbal Intelligence (NART)	112.6	(±8.2)	115.6	(±5.4)	109.2	(±9.1)	110.6	(±7.5)	4.1	.008	R>D
Duration of Formal Education (Years)	12.7	(±1.9)	13.4	(±1.7)	12.3	(±2.3)	11.6	(±1.7)	5.8	.001	R>D and C>D
Disposable Income (£ per Month)	629	(±911)	839	(±1208)	421	(±414)	370	(±622)	3.0	.032	R>D
Childhood Maltreatment (CTQ)^b^	17.8	(±5.5)	18.8	(±3.8)	24.3	(±10.7)	28.5	(±14.4)	12.3	<.001	D = S>R = C
Alcohol Consumption (AUDIT)	3.3	(±2.2)	5.7	(±1.5)	3.8 (±4.5)		11.1	(±11.1)	11.8	<.001	D>R = S = C
Drug-Taking Experiences (DAST-20)	.0	(±.0)	2.4	(±1.0)	.5	(±1.1)	not administered		81.5	<.001	R>S>C
Duration of Stimulant Use (Years)^c^			7.9	(±5.8)			16.1 (±6.4)		−5.5	<.001	
Age of Onset Stimulant Use (Years)^c^			20.2	(±4.8)			16.4	(±2.8)	4.4	<.001	
Age of Onset Cannabis Smoking (Years)	17.6	(±4.0) ^*n* = 11^	17.9	(±6.8) ^*n* = 26^	17.7	(±4.2) ^*n* = 35^	14.5	(±3.2) ^*n* = 50^	5.3	.002	D>R = S = C
Age of Onset Tobacco Smoking (Years)	16.0	(±2.8) ^*n* = 8^	16.1	(±4.0) ^*n* = 24^	14.5	(±2.0) ^*n* = 46^	12.5	(±3.3) ^*n* = 49^	12.2	<.001	D>R = S = C
Cigarette Consumption (Number/Day)^d^	7.1	(±5.5)	7.0	(±5.6)	5.0	(±7.8)	15.7	(±12.5)	5.2	.001	D>R = S = C

AUDIT, Alcohol Use Identification Test (cutoff score for harmful alcohol use:>8), Saunders *et al.*(24); CTQ, Childhood Trauma Questionnaire, Bernstein *et al.*(28); DAST-20, Drug Abuse Screening Test (cutoff score for harmful use:>5), Skinner (23); NART, National Adult Reading Test, Nelson (27).

a Abbreviations of the groups in the post hoc tests: C, control; R, recreational; S, sibling; D, dependent.

b Composite score of the subscales: emotional abuse, physical abuse, sexual abuse.

c Stimulant use includes the duration/onset of amphetamine, cocaine, and crack-cocaine use.

d Cigarette consumption refers to the numbers of cigarettes smoked by those individuals in the group who were smokers.
