# Supplementary material for: Distinctive Personality Traits and Neural Correlates Associated with Stimulant Drug Use Versus Familial Risk of Stimulant Dependence
Source: Biol Psychiatry. 2013 Jul 15;74(2):137–44. doi: 10.1016/j.biopsych.2012.11.016 (PMC3705207; doi:10.1016/j.biopsych.2012.11.016)
Supplement: Supplementary file 1 — Supplementary materials [file mmc1.pdf]

Distinctive Personality Traits and Neural Correlates Associated with Stimulant Drug Use versus Familial Risk of Stimulant Dependence

Supplemental Information

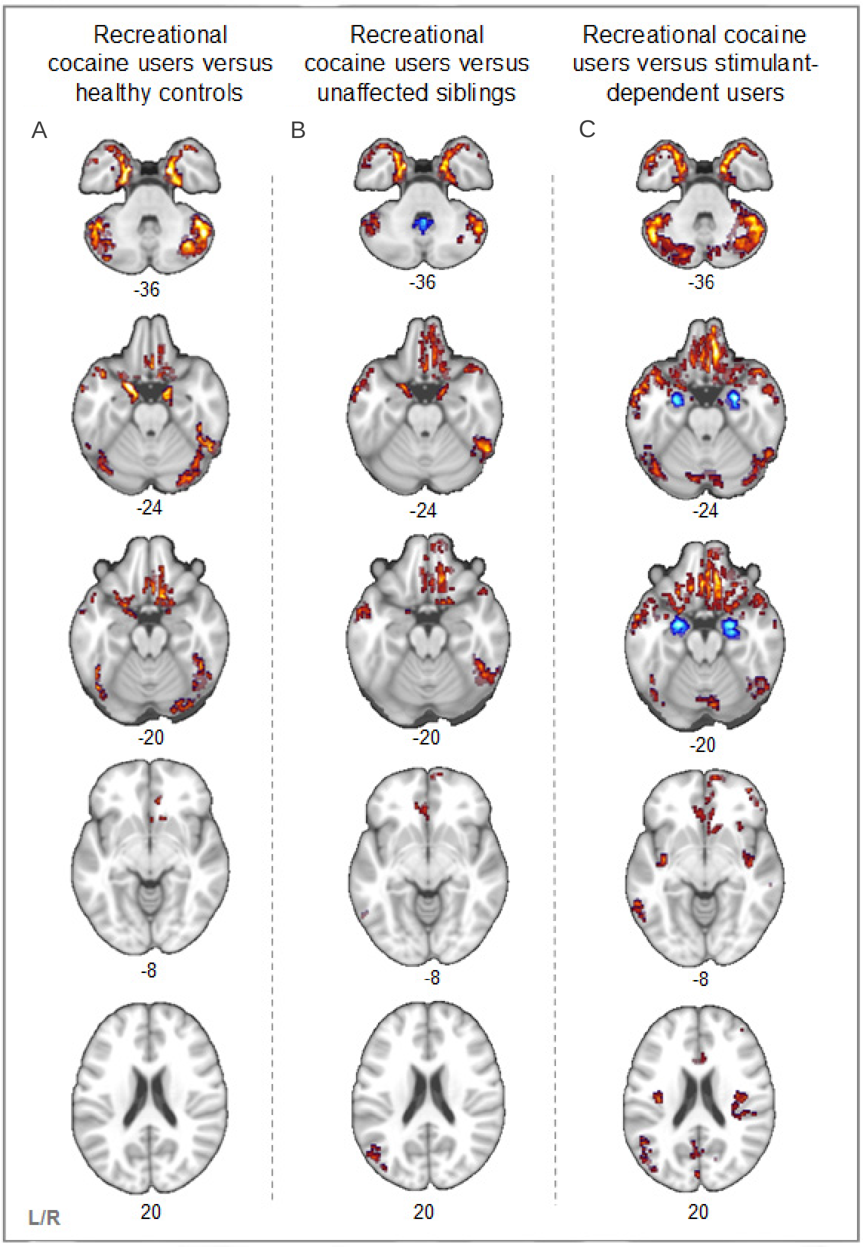

Figure S1. Structural abnormalities in (**A**) recreational cocaine users compared with control volunteers, (**B**) with the siblings of stimulant-dependent individuals, and (**C**) with the stimulant-dependent volunteers. Blue voxels indicate a decrease and red voxels indicate an increase in gray matter volume compared with controls.

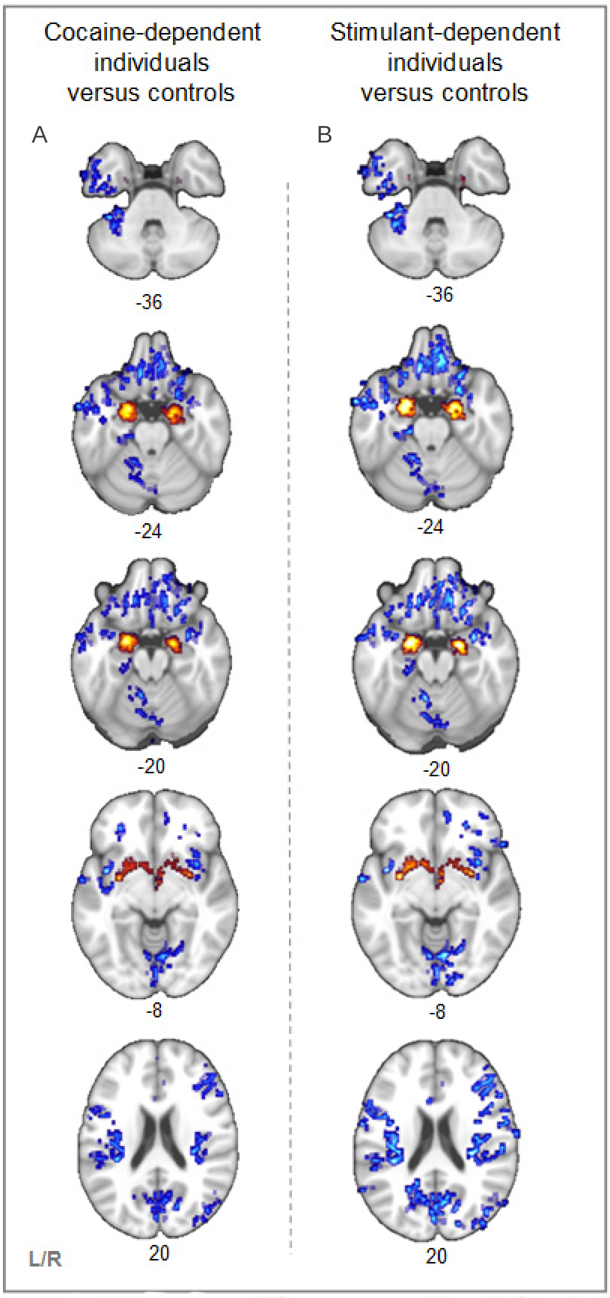

Figure S2. (**A**) All individuals in the sample who were dependent on cocaine were compared with control volunteers. (**B**) Gray matter volume in all stimulant-dependent individuals, including the three individuals who were dependent on amphetamines, was compared with healthy volunteers.
